# Multi-functional BST2/tetherin against HIV-1, other viruses and LINE-1

**DOI:** 10.3389/fcimb.2022.979091

**Published:** 2022-09-13

**Authors:** Yifei Zhao, Ke Zhao, Shaohua Wang, Juan Du

**Affiliations:** ^1^ Center of Infectious Diseases and Pathogen Biology, The First Hospital of Jilin University, Changchun, China; ^2^ Institute of Virology and AIDS Research, The First Hospital of Jilin University, Changchun, China

**Keywords:** BST2, tetherin, HIV-1, immunomodulation, LINE-1

## Abstract

Bone marrow stromal cell antigen 2 (BST2), also known as CD317, HM1.24, or tetherin, is a type II transmembrane glycoprotein. Its expression is induced by IFN-I, and it initiates host immune responses by directly trapping enveloped HIV-1 particles onto the cell surface. This antagonistic mechanism toward the virus is attributable to the unique structure of BST2. In addition to its antiviral activity, BST2 restricts retrotransposon LINE-1 through a distinct mechanism. As counteractive measures, different viruses use a variety of proteins to neutralize the function or even stability of BST2. Interestingly, BST2 seems to have both a positive and a negative influence on immunomodulation and virus propagation. Here, we review the relationship between the structural and functional bases of BST2 in anti-HIV-1 and suppressing retrotransposon LINE-1 activation and focus on its dual features in immunomodulation and regulating virus propagation.

## Introduction

**Figure 1:**
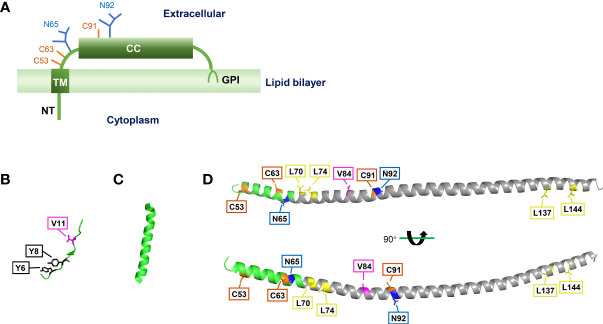
Structure of BST2. **(A)** Main features of BST2 structure. NT, N-terminal cytoplasmic tail; TM, transmembrane region; CC, extracellular coiled-coil domain; GPI, glycosyl-phosphatidylinositol anchor. **(B)** Crystal structure of BST2 NT domain, separated from crystal structure of the human BST2 cytoplasmic domain and the HIV-1 Vpu cytoplasmic domain bound to the clathrin adaptor protein complex 1 (AP1) core (Jia et al., 2014) (RCSB PDB code: 4P6Z). **(C)** Crystal structure of BST2 TM domain (Skasko et al., 2012) (RCSB PDB code: 2LK9). **(D)** Crystal structure of BST2 ectodomain (Schubert et al., 2010) (RCSB PDB code: 3NWH). The CC domain is labeled in gray. These structures were re-constructed with the PyMOL software (version 2.5.3). Specific amino acid residues are highlighted as follows: tyrosine residues = black boxes; valine residues = pink boxes; disulfide bonds = orange boxes; N-linked glycosylation sites = blue boxes; leucine residues = yellow boxes.

Bone marrow stromal cell antigen 2 (BST2), also known as tetherin, HM1.24, or CD314, is a type II transmembrane glycoprotein. The *BST2* gene, located on chromosome 19p13.2, was first cloned from a human rheumatoid arthritis-derived synovial cell line (J Ishikawa et al., 1995). However, BST2 is now known to be expressed in many types of cells, such as T cells and plasmacytoid dendritic cells. It was initially described as a novel marker of terminally differentiated B cells, which could be recognized by an anti-HM1.24 monoclonal antibody (Goto et al., 1994; Ohtomo et al., 1999). However, querying databases with the cDNA sequence revealed that BST2 and HM1.24 were the same protein (Ohtomo et al., 1999). Human BST2 contains 180 amino acids, yet diffuse bands of 29–33 kDa are normally detected in SDS-PAGE due to protein glycosylation (Goto et al., 1994). BST2 consists of a short N-terminal cytoplasmic (NT) domain, an α-helical transmembrane (TM) domain, an extracellular domain (ED) containing an α-helical coiled-coil (CC) domain, and a C-terminal glycosyl-phosphatidylinositol (GPI) anchor (Hinz et al., 2010; Schubert et al., 2010; Yang et al., 2010) (**Figure 1A**).

As a host restriction factor, BST2 inhibits human immunodeficiency virus (HIV)-1 reproduction by physically trapping budding virions (it was termed “tetherin” for its ability to tether them to the plasma membrane) (Van Damme et al., 2008; Neil et al., 2008) and regulating host immune responses (Galao et al., 2012; Tokarev et al., 2013). BST2 can also inhibit the LINE-1 retrotransposon by regulating the promoter activity of 5′UTR (Zhao et al., 2021). Surprisingly, BST2 is indispensable for the infection, proliferation, and transmission of certain viruses, including HIV-1 (Viswanathan et al., 2011; Olety et al., 2021). In this review, we discuss the relationship between the structure and functions of BST2 and the latest findings on the protein.

## Structure of BST2

**Figure 2:**
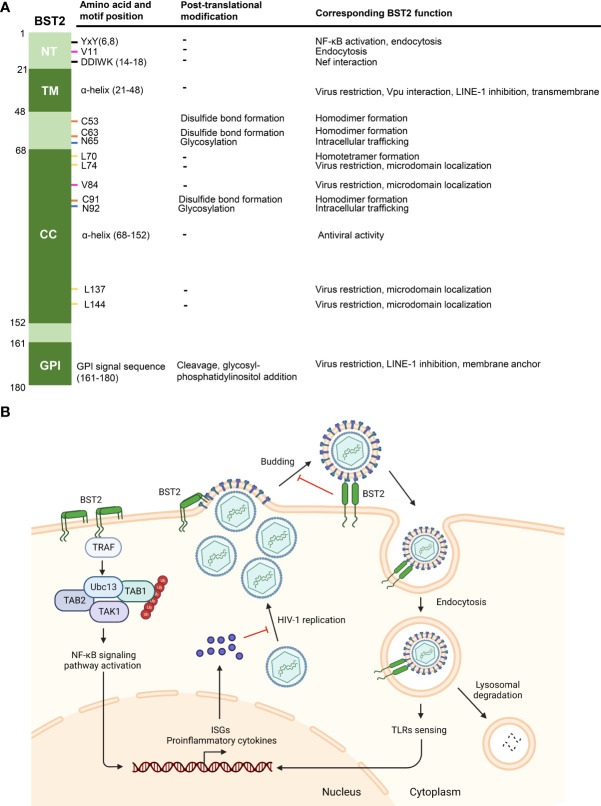
Functions of BST2. **(A)** Post-translational modifications and corresponding functions of BST2 domains and amino acid residues. Specific motifs and residues (amino acid and motif position, first column) implicated in different post-translational modifications (second column) are listed. The roles of motifs and modifications of each residue are listed under ‘corresponding BST2 function’. **(B)** The diagram showing known anti-HIV-1 functions of BST2. BST2 tethers budding HIV-1 particles to the cell membrane, and the tethered viruses may be internalized and degraded in lysosome. Endocytic vesicles containing BST2 and HIV-1 particles activate ISGs and proinflammatory cytokines expression through TLRs sensing, which then inhibits HIV-1 replication. On the other hand, clustering of BST2 dimers induces the activation of NF-κb signaling pathway and subsequent proinflammatory responses. Panel B was created with BioRender.com.

Although the crystal structures of NT, TM, and ED domains of BST2 have been reported (Swiecki et al., 2011; Skasko et al., 2012; Jia et al., 2014) (**Figures 1B-D**), the complete crystal structure of full-length BST2 remains to be revealed in future research. Yet, previous studies have uncovered has several key structural features about BST2 (**Figure 2A**). First, as a raft-associated apical membrane protein, BST2 contains two membrane-associated domains: TM and GPI. TM contains a signal sequence that directs nascent BST2 to the rough endoplasmic reticulum (RER). As a result, TM is inserted into the ER membrane, and the C-terminal nascent peptide is in the lumen. This is important for the extracellular exposure of the CC domain, essential for the antiviral function of BST2. In addition, BST2 uses the GPI anchor to accumulate in cholesterol-rich lipid microdomains (i.e., lipid rafts) at the trans-Golgi network (TGN) and plasma membrane (Kupzig et al., 2003; Rollason et al., 2007). As a key step in BST2 modification, the C-terminal signal for GPI addition is cleaved and the GPI portion is transferred to luminal domain of BST2. GPI modification is altered in the absence of these enzymes, leaving BST2 incompletely processed and unable to exit the ER, indicating that ER modification of the GPI anchor is required for proper BST2 intracellular transport (Perez-Caballero et al., 2009; Maeda and Kinoshita, 2011).

BST2 also contains two conserved domains: NT and CC. NT is the only intracellular component of BST2, suggesting a possible cytoplasmic function. The NT domain contains a dual-tyrosine-based internalization signal that allows BST2 to continuously cycle between the plasma membrane and the TGN through early and recycling endosome membranes (Kupzig et al., 2003; Rollason et al., 2007; Masuyama et al., 2009). However, it remains unclear whether the internalization signal contains two independent YxxØ motifs that mediate clathrin-dependent internalization, or the internalization motif is a single one that requires two Y residues (Rollason et al., 2007). NT is involved in innate immune regulation, and its highly conserved YxY motif is important in NF-κb activation, and for association with the actin cytoskeleton (Galao et al., 2012; Tokarev et al., 2013; Galão et al., 2014). The CC domain, with its seven-amino acid repeat, occupies most of the ED. However, CC domains contribute little to the production and conformation of BST2. A previous study suggested that the CC domain may contribute to BST2 dimerization, likely through two salt bridges and an interhelical hydrogen bond (Hinz et al., 2010). However, these weak interactions are not enough to maintain BST2 dimers without the formation of three cysteine disulfide bonds. In contrast, the ectodomain appears to have multiple functions in HIV-1 particle restriction, which will be discussed later.

Furthermore, the ED contains several amino acids essential for the proper structure and localization of BST2. The three cysteine residues mentioned above are critical for disulfide bond formation between two adjacent BST2 proteins, and these bonds are formed between the same cysteine residues on each BST2 (i.e., C53–C53, C63–C63, and C91–C91), thus forming parallel dimers (Yang et al., 2010; Arias et al., 2011). Zinc inhibits the dimerization of BST2 by binding cysteine residues, thus affecting its antiviral activity (Petrosino et al., 2021). Another essential residue for BST2 polymerization is L70, which allows the formation of homotetramers (Schubert et al., 2010). It is largely dispensable for the restriction of virion release but required for the induction of NF-κb (Tokarev et al., 2013).

N65 and N92, which are two other BST2 residues are involved in BST2 transportation but not polymerization. These two asparagine sites are first glycosylated in the ER, allowing BST2 to be transported to the TGN, where the glycosylation is further modified so that BST2 can finally be transported to the cytoplasmic membrane (Fujita et al., 2012). Mutating these two residues can abolish BST2 glycosylation, effectively sequestering BST2 in the ER (Perez-Caballero et al., 2009).

In short, BST2 is a transmembrane protein with several intriguing features: 1) it is synthesized in the RER and transported to the cytoplasmic membrane; 2) it has two separated membrane-associated domains; 3) it can form homodimers and homotetramers; 4) it cycles between the cytoplasmic membrane and the TGN. This review aims to assess how these features contribute to BST2 biological functions.

## Classical function of BST2: Trapping HIV-1

The most well-known function of BST2 is to tether newly-synthesized, enveloped-virions onto the cytoplasmic membrane of the host cell (**Figure 2B**). This was first proposed to explain BST2-mediated HIV-1 inhibition (Van Damme et al., 2008; Neil et al., 2008). As opposed to other antiviral factors (e.g., APOBEC3 proteins) that suppress HIV through enzymatic activities (Salter et al., 2016), BST2 tethers HIV-1 virions through a physical link between the two, preventing the release of newly-formed budding HIV-1 particles (Perez-Caballero et al., 2009; Hinz et al., 2010). This cell-to-virus connection can be disrupted with proteases, confirming this physical effect (Neil et al., 2006; Neil et al., 2008). In fact, BST2 has been shown to inhibit a diverse range of viruses in addition to HIV-1, including other retroviruses (HIV-2, SIV, HTLV-I, MLV) (Jouvenet et al., 2009; Le Tortorec and Neil, 2009), Ebola virus (Jouvenet et al., 2009), Hepatitis B virus (Yan et al., 2015), CHIKV (Jones et al., 2013) and SARS-Cov-2 (Petrosino et al., 2021).

It is well-known that protein function is determined by structure, and BST2 is one of the best exemplars of this theory. Almost all conserved domains and critical residues mentioned above are involved in the BST2 “tethering” function. Both TM and GPI are indispensable for BST2-induced HIV-1 restriction, as removal of either end abrogates the BST2 antiviral effect (Perez-Caballero et al., 2009). At present, the widely accepted model is that the parallel dimer TM domain formed by BST2 is fixed in the host cell, and the GPI anchor is inserted into the envelope of HIV-1, so as to physically inhibit the release of virus particles (Venkatesh and Bieniasz, 2013). However, the detailed mechanism under such a phenomenon (e.g., how GPI recognizes HIV particle and how TM maintains its position on the cytoplasmic membrane) is to be revealed. Notably, they are the only two membrane-associated domains in the protein, indicating that membrane insertion is essential for BST2-mediated virion tethering. This explains why BST2 targets enveloped viruses such as HIV.

ED-mediated homodimerization is essential in virion tethering and immunomodulation. Although weak CC domain interactions contribute little to BST2 dimerization, cysteines (C53, C63, and C91) are responsible for intermolecular disulfide bonds resulting in homodimerization, critical for HIV-1 restriction (Andrew et al., 2009; Yang et al., 2010). Further, the two N-linked glycosylation sites, N65 and N92, are indispensable for BST2 tethering activity, as BST2 mutants lacking glycosylation sites are sequestered in the ER and cannot assemble at the lipid rafts (HIV-1 budding sites) (Perez-Caballero et al., 2009; Hu et al., 2012). Although deletion of most of the CC domain strongly inhibited antiviral activity (Perez-Caballero et al., 2009), the CC structure offers high plasticity in restricting HIV-1 particle release. Four key N-and C-terminal positions (L74, V84, L137, and L144) are critical for BST2 membrane microdomain localization and particle-release restriction, as the substitution of polar serine residues for hydrophobic residues affect nearby N65 and N92 glycosylation (Hammonds et al., 2012). Except for these four sites, mutations or truncations induced in most other ED regions, including removal of all seven heptad motifs thought to be critical for CC structure formation, fail to disrupt BST2 tethering ability (Hammonds et al., 2012; Andrew et al., 2012). These data demonstrate that, except for special sites, the size and primary sequence of the CC domain shows substantial flexibility in HIV-1 restriction, while this conserved coiled-coil structure may serve other functions in HIV-1 inhibition. The CC domain is the only region completely exposed to the extracellular environment; thus, it may interact with currently unknown extracellular ligand to trigger an intracellular antiviral signaling pathway. Further study is required to verify such a hypothesis.

Although BST2 can inhibit virion release, the fate of the mature, cell surface-tethered virions are not known. These virions either are endocytosed and transported for degradation or enhance cell-to-cell spread of infection. The trapped virions-BST2 complex is proposed to be endocytosed *via* interaction with actin-binding protein lymphocyte-specific protein 1 (LSP1) (Kulkarni et al., 2020). Based on structural analysis and reasonable assumption, the LSP1/BST2 interaction would be dependent on the LSP1 N-terminal domain and YxY motif of BST2, known to interact with the actin cytoskeleton (Mahauad-Fernandez and Okeoma, 2016; Kulkarni et al., 2020). After internalization, most of the HIV-1 is degraded in lysosomes, with the remaining minority retained in endocytic compartments and depleted through the proteasome pathway (Smith et al., 2007). Cell-to-cell virion spreading will be discussed later.

## Dual immunomodulatory role of BST2

In addition to virion tethering, BST2 can respond to infection by HIV-1 or other enveloped viruses by inducing proinflammatory responses *via* NF-κb signaling pathway activation (Galao et al., 2012; Tokarev et al., 2013). Upon viral invasion, pattern recognition receptors (PRRs) initiate the innate immune response and trigger the production and activation of type 1 interferons (IFNs) and proinflammatory cytokines. IFN-I activates the corresponding receptors and induces the expression of hundreds of IFN-stimulated genes (ISGs) to limit virus replication and spreading (Schneider et al., 2014; Schlee and Hartmann, 2016). As a host restriction factor, BST2 expression is regulated by IFN-I and other cytokine signaling (Schneider et al., 2014), and BST2 triggers a positive-feedback pathway to induce IFN-I expression in return. On the cell surface, BST2 promotes the recruitment of a signaling complex through the YxY (Y6,8) motif on its NT domain, leading to TGF β-activated kinase 1 (TAK1) and NF-κb activation and enhanced proinflammatory gene expression (Galao et al., 2012; Tokarev et al., 2013). Other intermediate signaling molecules in NF-κb signal pathway activation (TNF receptor associated factor 6 (TRAF6), TRAF2, ubiquitin-conjugating enzyme E2N (Ubc13), etc.) were successively identified as components of this BST2-recruited signaling complex (Galao et al., 2012; Tokarev et al., 2013) (**Figure 2B**). Interestingly, the mRNA of BST2 also encodes a short isoform (BST2-S) by alternative translation, which misses the N-terminal 12 residues, including the YxY motif, in its cytoplasmic tail. Consequently, BST2-S does not activate NF-κb (Cocka and Bates, 2012). BST2 dimerization and tetramerization are also important for NF-κb activation, as substitutions in C53, C63, C91, and L70 inhibited protein-induced NF-κb activation (Tokarev et al., 2013). TLRs or other PRRs recognize the endocytic compartments containing BST2 and trapped HIV-1 virions, indirectly inducing IFN-I responses in infected primary cells (Galao et al., 2012). Although the components of the recruitment complex vary in different studies, BST2 is an irreplaceable element that positively regulates the host inflammatory response.

IFN overexpression may cause multiple severe autoimmune diseases (Gota and Calabrese, 2003). Therefore, IFN regulation is essential. BST2 can suppress the IFN-I response by degrading the mitochondrial antiviral-signaling protein (MAVS), important for retinoic acid-inducible gene I (RIG-I)-like receptor (RLR)-mediated IFN- I signal response (Jin et al., 2017). BST2 recruits E3 ubiquitin ligase MARCH8 to mediate K27-linked MAVS ubiquitination, attracting nuclear domain 10 protein 52 (NDP52) to trigger lysosomal degradation of MAVS (Jin et al., 2017). Furthermore, BST2 interacts with ILT7 and strongly inhibits IFN-I and proinflammatory cytokine production in plasmacytoid dendritic cells (pDCs) (Cao et al., 2009). BST2 also suppresses IFN-I promoter activity by compromising LINE-1 retrotransposition (Zhao et al., 2021). LINE-1 RNP formation allows LINE-1 RNA to trigger IFN production through melanoma differentiation-associated protein 5 (MDA5) and RIG-I-mediated RNA-sensing pathways (Zhao et al., 2018). BST2 employs these pathways to suppress IFN-I promoter activation by decreasing LINE-1 RNA production (Zhao et al., 2021). These findings present BST2 as a bi-directional controller in immunomodulation. BST2 induces NF-κb-dependent proinflammatory gene expression right after viral infection and prevents prolonged IFN production in a negative-feedback manner. This switch in regulation may help to prevent host cells from over-activating the autoimmune system. Additional research is required to determine how BST2 changes its role to affect the innate antiviral immune response at the appropriate time.

## Role of BST2 in LINE-1 retrotransposition

Retrotransposons in mammals are ancient retroviruses inserted within germ cells during evolution. LINE-1 is the only known type that can replicate autonomously (Ostertag and Kazazian, 2001). Similarities between the replication processes of retrotransposon LINE-1 and retrovirus HIV-1 indicate that several host restriction factors with confirmed anti-HIV-1 activity may also regulate LINE-1 retrotransposition (Zhao et al., 2021). BST2 suppresses LINE-1 activity, but the underlying mechanism remains unknown (Goodier et al., 2015). Since HIV-1-trapping by BST2 takes place on the cell surface and LINE-1 replication takes place in the cell, we believe that the LINE-1 mechanism of inhibition by BST2 is different from budding-HIV-1 restriction. We have confirmed LINE-1 retrotransposition suppression *via* BST2-mediated decrease of both LINE-1 protein and RNA levels by regulating LINE-1 5′UTR promoter activity (Zhao et al., 2021). BST2 also activates the NF-κb signaling pathway through signaling motifs in its NT domain, regulating a series of downstream ISG promoter activities (Galao et al., 2012; Tokarev et al., 2013). However, the LINE-1-suppressing activity is not affected by BST2 lacking the NT domain, implying that the BST2-mediated mechanism of LINE-1 inhibition is unrelated to its NF-κb signaling pathway-inducing function (Zhao et al., 2021). BST2 may interact with other membrane proteins through its two transmembrane regions and then activate other signaling pathways to inhibit LINE-1 retrotransposition, which requires further research to confirm.

Under normal conditions, only 80–120 LINE-1 copies out of ~500,000 per human cell retain retrotransposition activity, and abnormally activated LINE-1 can cause severe DNA damage and autoimmune diseases. BST2-mediated downregulation of LINE-1 retrotransposition activity protects the stability of genomic DNA and decreases innate immune activation by reducing LINE-1 RNA levels (Zhao et al., 2021). This highlights the biological significance of BST2 in the absence of infection and provide a new mode by which BST2 may participate in immune response suppression.

## HIV-1 antagonistic action and mechanism against BST2

To overcome inhibition by BST2, viruses have developed various specific countermeasures. The most well-known is the HIV-1 protein Vpu, a transmembrane protein localized in the plasma membrane, ER, TGN, and endosomal compartments of host cells. Vpu impairs BST2 to enhance HIV-1 release and pathogenesis through two different mechanisms. The first is sequestering BST2 away from the plasma membrane where virions bud (Van Damme et al., 2008; Chu et al., 2012) by direct interaction between the BST2 and Vpu transmembrane domains, thus altering BST2 trafficking and recycling pathways between the cell surface and intracellular compartments (Dube et al., 2009; Schmidt et al., 2011; Mcnatt et al., 2013; Jia et al., 2014). Another mechanism is decreasing BST2 levels by degradation after downregulating BST2 at virion budding sites. The Vpu-BST2 complex triggers BST2 ubiquitination by recruiting the E3 ubiquitin ligase adaptor β-TrCP through the Vpu DSGXXS motif (Douglas et al., 2009; Mitchell et al., 2009; Mangeat et al., 2009). This E3 ubiquitin ligase complex facilitates the ubiquitination of the BST2 NT region, leading to the lysosomal degradation of BST2. BST2-S, however, is insensitive to Vpu antagonism, since it lacks the ubiquitin acceptor sites (Douglas et al., 2009; Iwabu et al., 2009; Caillet et al., 2011; Janvier et al., 2011; Cocka and Bates, 2012).

Only some lineages of two major primate lentiviruses (HIV-1 and several SIVs) express Vpu. However, many other viruses utilize different viral proteins and antagonistic mechanisms to repress BST2. HIV-2 and several SIVs employ Env to downregulate BST2 on the cell surface, promote BST2 endocytosis, and sequestrate BST2 in the TGN without enhanced degradation (Gupta et al., 2009; Le Tortorec and Neil, 2009; Serra-Moreno et al., 2011). This BST2 antagonism requires a conserved, tyrosine-based endocytosis motif in the membrane-proximal region of the gp41 tail (Noble et al., 2006; Le Tortorec and Neil, 2009). SIV strains, such as SIVagm, SIVblu, and SIVmac, lack the *vpu* gene and use the viral accessory protein Nef to counteract BST2. Even the SIV strains SIVgor and SIVcpz, capable of expressing Vpu, rely on Nef to counteract BST2 (Jia et al., 2009; Sauter et al., 2009; Zhang et al., 2009). A direct BST2-Nef interaction separates BST2 from virus-release sites at the plasma membrane through endocytosis and promotes BST2 lysosomal degradation (Serra-Moreno et al., 2013). The M group HIV-1 strain AD8, unable to express Vpu owing to a mutation, utilizes Nef to enhance BST2 internalization and perinuclear accumulation without BST2 degradation (Giese et al., 2020). Although the specific mechanism is not fully known, the available results show that neither Env nor Nef can decrease BST2 through the ubiquitin-proteasome system, suggesting that BST2 presents a barrier to initial HIV transmission to humans. Vpu expression was likely a means to overcome this obstacle, thereby facilitating the human HIV pandemic.

## HIV-1 and other viruses utilize BST2 in propagation

Some viruses have developed patterns that use BST2 to increase their propagation by promoting replication, entry, and cell-to-cell spreading. Basal BST2 levels are necessary for propagation of HIV-1 in immune cells, including MOLT-3, Jurkat, and PBMC cells and of measles virus (MV) in mice brains and primary cultured neurons (Olety et al., 2021; Kdma et al., 2021). The underlying mechanism is mostly unknown, except that BST2 acts in a structure-dependent manner in HIV-1 replication. BST2 activates the NF-κb signaling pathway, and NF-κb p50/p65 heterodimers bind to the enhancer region in the long terminal repeat of HIV-1 and initiate HIV-1 transcription (Yu Mingyan, 2009; Galao et al., 2012); thus, HIV-1 propagation may be dependent on BST2-mediated NF-κb pathway activation. This hypothesis was also confirmed to some extent by the impaired production of SARS-CoV, SARS-CoV-2, and avian influenza virus in the presence of the BST2 mutant lacking both CT and TM domains (Dolskiy et al., 2020). In addition, it is also reasonable to assume that BST2 can facilitate MV and HIV-1 replication through a mechanism related to BST2 suppression of LINE-1 promotor activity. Thus, BST2 as a well-known antiviral factor has shown potentials to be a therapeutic target against viral production.

BST2 tether action can also accumulate virions on the cell surface, increasing the opportunities for the trapped virus to contact nearby cells. This tether-promoted cell-to-cell spreading has been observed in several viruses, including HIV-1, feline immunodeficiency virus (FIV), and influenza A (Jolly et al., 2010; Dietrich et al., 2011; Londrigan et al., 2015). Other viruses, such as cytomegalovirus (HCMV), vesicular stomatitis virus (VSV), and influenza B, utilize BST2 functions to enter the cells (Viswanathan et al., 2011; Swiecki et al., 2012). BST2 expression increases HCMV entry by interacting with HCMV particles on the cell surface *via* a reverse-tethering mechanism in human monocytes and fibroblasts (Viswanathan et al., 2011). BST2-KO mice infected with VSV or influenza B also show decreased viral loads *in vivo* (Swiecki et al., 2012). Thus, BST2 tethering may play a dual role, suppressing viral production or release, or helping viruses to capture cells and facilitating viral entry. This may also be another HIV-1 strategy to balance virus production decrease, independently of Vpu-mediated neutralization. The fact that host restriction factors can be both antagonized and exploited by viruses may be more complicated than anticipated, deserving further investigation of the underlying mechanisms and equilibrium conditions.

## Discussion

BST2 is an important component of the barrier against HIV-1 and other viruses. Its antiviral roles can be divided into physical tethering and immunomodulation; however, it appears that both effects are two-sided. Tethering can inhibit virus release, leading to endocytosis, but this can also be used by viruses to capture host cells, facilitating their entry. Regarding the bi-directional role of BST2 in immunoregulation, we speculate that it is a protective measure of the host immune system to prevent overactive autoimmunity, rather than viruses utilizing BST2 to reduce immune activation to escape the host immune response. In either case, further studies are necessary to elucidate how BST2 switches modes to achieve bi-directional immune response regulation.

The NF-κb signaling pathway triggers ISGs expression in an IFN-independent manner. This function requires the YxY motif in the NT domain of BST2. Conversely, BST2 suppresses LINE-1 retrotransposition by compromising LINE-1 5′ UTR promoter activity, where the YxY motif most likely plays no role. We have discovered other gene promoters (including HCMV and several endogenous gene promoters) promoted or inhibited by BST2 (Zhao et al., 2021). This hints at the possibility of BST2 regulating viral protein expression and may uncover the role of BST2 in other physiological conditions, such as tumor development. A deeper understanding of BST2-mediated promoter regulation may inform the design of innovative therapeutic approaches against exogenous viral infections and endogenous conditions like autoimmune diseases and cancer.

## Author contributions

JD chose the topic. All authors discussed and drafted the manuscript. All authors contributed to the article and approved the submitted version.

## Funding

This work was supported in part by grants from National Natural Science Foundation of China [32170146, 32170140, 81902049, and 32100108]; Science and Technology Department of Jilin Province [20220101301JC, 20220101285JC, 20200201525JC]; China Postdoctoral Science Foundation [2020M670843]; Fundamental Research Funds for the Central Universities [2017TD-08]; Key Laboratory of Molecular Virology, Jilin Province [20102209]; National Natural Science Foundation of Jilin Province [JLSCZD2019-008]; Norman Bethune Health Science Center of Jilin University [2018B18], and First Hospital of Jilin University [2020-CXQ-02].

## Conflict of interest

The authors declare that the research was conducted in the absence of any commercial or financial relationships that could be construed as a potential conflict of interest.

## Publisher’s note

All claims expressed in this article are solely those of the authors and do not necessarily represent those of their affiliated organizations, or those of the publisher, the editors and the reviewers. Any product that may be evaluated in this article, or claim that may be made by its manufacturer, is not guaranteed or endorsed by the publisher.
